# Vascular smooth muscle cell-derived nerve growth factor regulates sympathetic collateral branching to innervate blood vessels in embryonic skin

**DOI:** 10.1242/bio.060147

**Published:** 2024-05-21

**Authors:** Wenling Li, Katherine Lipsius, Nathan G. Burns, Ryo Sato, Azaan Rehman, Hui Xue, Christian Combs, Liliana Minichiello, Harshi Gangrade, Emmanouil Tampakakis, Yoh-suke Mukouyama

**Affiliations:** ^1^ Laboratory of Stem Cell and Neuro-Vascular Biology, Cell and Development Biology Center, National Heart, Lung, and Blood Institute, National Institutes of Health, Bethesda, MD 20892, USA; ^2^ Imaging AI Program, National Heart, Lung, and Blood Institute, National Institutes of Health, Bethesda, MD 20892, USA; ^3^ Light Microscopy Core, National Heart, Lung, and Blood Institute, National Institutes of Health, Bethesda, MD 20892, USA; ^4^ Department of Pharmacology, University of Oxford, Oxford OX1 4BH, UK; ^5^ Division of Cardiology, Johns Hopkins School of Medicine, Baltimore, MD 21218, USA

**Keywords:** Sympathetic axons, Collateral branching, Vascular smooth muscle cells, Nerve growth factor

## Abstract

Blood vessels serve as intermediate conduits for the extension of sympathetic axons towards target tissues, while also acting as crucial targets for their homeostatic processes encompassing the regulation of temperature, blood pressure, and oxygen availability. How sympathetic axons innervate not only blood vessels but also a wide array of target tissues is not clear. Here we show that in embryonic skin, after the establishment of co-branching between sensory nerves and blood vessels, sympathetic axons invade the skin alongside these sensory nerves and extend their branches towards these blood vessels covered by vascular smooth muscle cells (VSMCs). Our mosaic labeling technique for sympathetic axons shows that collateral branching predominantly mediates the innervation of VSMC-covered blood vessels by sympathetic axons. The expression of nerve growth factor (NGF), previously known to induce collateral axon branching in culture, can be detected in the vascular smooth muscle cell (VSMC)-covered blood vessels, as well as sensory nerves. Indeed, VSMC-specific *Ngf* knockout leads to a significant decrease of collateral branching of sympathetic axons innervating VSMC-covered blood vessels. These data suggest that VSMC-derived NGF serves as an inductive signal for collateral branching of sympathetic axons innervating blood vessels in the embryonic skin.

## Teaser

**Summary:** VSMC-derived NGF controls sympathetic collateral branching.

## INTRODUCTION

The vertebrate sympathetic nervous system is an essential series of reactionary and reciprocal processes that mediate physiological actions involved in the regulation of homeostasis. These include the control of body temperature, blood pressure, heart rate, blood glucose released from the liver, and the dilation of lung bronchioles achieved through the relaxation of airway smooth muscle (Goldstein, 2013). The proper regulation of homeostatic processes by the sympathetic nervous system is contingent upon the appropriate innervation of various tissues during development. Sympathetic neurons originate from sympathetic ganglia (SG) and extend their axons along blood vessels over considerable distances to establish connections with various tissue targets. Impaired sympathetic innervation and its dysfunction are associated with various cardiovascular diseases, including hypertension and hypotension (Scott-Solomon et al., 2021). In mice, the deletion of the homeobox transcription factor *Phox2b* results in the failure of proper formation and subsequent degeneration of all autonomic ganglia (Pattyn et al., 1999). This indicates the critical role of Phox2B in mediating sympathetic development. Indeed, *Phox2b* homozygous mutant embryos lack autonomic innervation to the heart and die *in utero* (Mokshagundam et al., 2021; Nam et al., 2013). While sympathetic axons innervate various tissue targets, including blood vessels, the mechanisms that regulate tissue-specific innervation are not yet completely understood.

Previous studies demonstrated that artemin, neurotrophin-3 (NT-3), nerve growth factor (NGF), and endothelin-3 (ET-3) are expressed by vascular smooth muscle cells (VSMCs) of blood vessels and stimulate sympathetic axon extension along blood vessels (Honma et al., 2002; Kuruvilla et al., 2004; Makita et al., 2008; Nam et al., 2013), while NGF is critical for controlling sympathetic innervation of final tissue targets (Glebova and Ginty, 2004; Shelton and Reichardt, 1984). Blood vessels serve not only as intermediate pathways that guide sympathetic axons towards their final targets but also represent targets for sympathetic innervation themselves. Indeed, after birth in mice, VSMC-derived netrin-1 is responsible for mediating arterial innervation in both mesenteric and skin vasculature (Brunet et al., 2014). However, the precise patterns and timing of sympathetic axon innervation to blood vessels, as well as the persistence of some axons along blood vessels towards their final targets, are still unclear. Furthermore, the mechanisms governing the process of sympathetic innervation to blood vessels during development remain incompletely understood.

To investigate the precise patterns and timing of sympathetic axon innervation to blood vessels, we employed a mouse embryonic limb skin vasculature model. We have previously demonstrated that in the embryonic limb skin, sensory nerves determine the pattern of large-diameter blood vessel branching, with VSMC coverage of these blood vessels observed by embryonic day (E) 15.5 (Li et al., 2013; Mukouyama et al., 2005, 2002). On one hand, *Neurogenin1/Neurogenin2 (Ngn1/Ngn2)* double homozygous mutants, lacking peripheral sensory nerves, demonstrate a pronounced absence of peripheral nerves and defective blood vessel branching in the limb skin at E15.5 (Mukouyama et al., 2002). On the other hand, *Phox2b* homozygous mutants, lacking peripheral autonomic nerves, exhibit the appropriate co-branching of peripheral nerves and blood vessels in the limb skin (Mukouyama et al., 2002). Together, these findings strongly suggest that sensory nerves, rather than sympathetic nerves, control blood vessel branching to form the co-branching of peripheral nerves and blood vessels in the limb skin. In this study, our time-course analysis with different embryonic stages in mice (E13.5-E18.5) has revealed that following the establishment of co-branching between sensory nerves and blood vessels, sympathetic axons invade the skin alongside sensory nerves, and subsequently they extend their branches towards these blood vessels. Our mosaic labeling technique for sympathetic axons has revealed that collateral branching, in which daughter branches sprout from the main nerve, predominantly mediates the innervation of blood vessels by sympathetic axons. As NGF is recognized for its role in promoting collateral branching *in vitro* (Armijo-Weingart and Gallo, 2017; Ketschek et al., 2015) and regulating final target innervation *in vivo* (Kuruvilla et al., 2004), our genetic studies in mice with VSMC-specific *Ngf* knockout unequivocally illustrate the crucial role of NGF derived from VSMCs in facilitating collateral branching of sympathetic axons within blood vessels. This data suggests that the expression of NGF in VSMCs is important to induce the formation of sympathetic collateral branching, specifically for their innervation of blood vessels in the embryonic skin.

## RESULTS

### Sympathetic axons extend their branches towards blood vessels, following the establishment of co-branching between sensory nerves and blood vessels

**Fig. 1.**
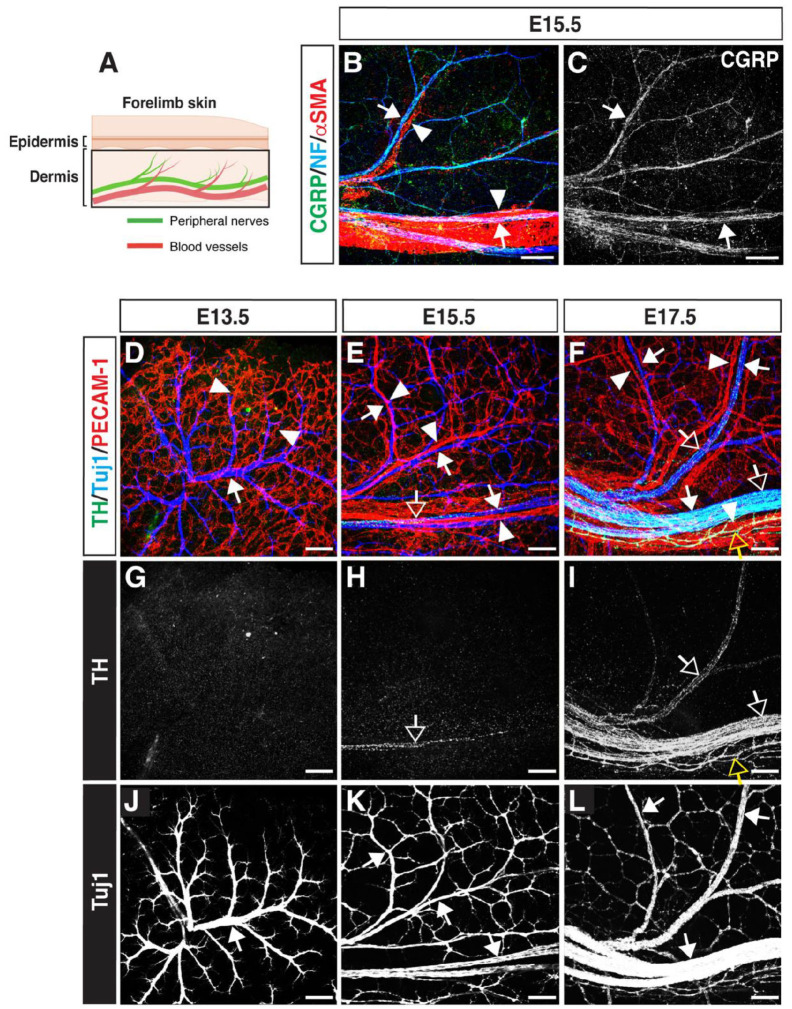
**Sympathetic axons extend their branches in the dermis of the skin, following the establishment of co-branching between sensory nerves and blood vessels.** (A) Schematic diagram illustrating the embryonic forelimb skin (created with BioRender.com). (B-C) Whole-mount triple immunofluorescence labeling of the dermis of the forelimb skin from E15.5 wild-type embryos with antibodies to the sensory neuron marker CGRP (green in B; white in C), the pan-neuronal cytoskeletal marker neurofilament (NF; blue in B), and the VSMC marker αSMA (red in B) are shown. Arrows indicate representative CGRP^+^/NF^+^ sensory nerves, and arrowheads indicate representative αSMA^+^ VSMC-covered blood vessels. Scale bars: 100 µm. (D-L) Whole-mount triple immunofluorescence labeling of the dermis of the forelimb skin from E13.5 (D, G and J), E15.5 (E, H and K) and E17.5 (F, I and L) wild-type embryos with antibodies to the sympathetic neuron marker tyrosine hydroxylase (TH; green in D-F; white in G-I), the pan-neuronal cytoskeletal marker class III ß-tubulin (Tuj1; blue in D-F; white in J-L), and the pan-endothelial cell marker PECAM-1 (red in D-F) is shown. Open arrows indicate representative TH^+^ sympathetic axons, arrows indicate representative Tuj1^+^ peripheral nerves, and arrowheads indicate representative capillaries at E13.5 (D) and medium- or large-diameter blood vessels at E15.5 and E17.5 (E and F). Scale bars: 100 µm. Note that certain TH^+^ sympathetic axons (yellow open arrows in F and I) extend their branches towards blood vessels at E17.5.

We performed whole-mount immunohistochemical analysis of the dermis of the forelimb skin from embryos at different stages (Fig. 1A). Our previous studies demonstrated that by E13.5, sensory nerves invade the dermis of the limb skin, where a primary capillary plexus has formed, and by E15.5, the primary capillary plexus remodels to form medium- or large-diameter arteries covered by vascular smooth muscle cells (VSMCs), which align with the pattern of sensory nerves (Li et al., 2013; Mukouyama et al., 2005, 2002). Indeed, the vessel-aligned nerves express the sensory neuron marker calcitonin gene-related peptide (CGRP) (Fig. 1B and C, arrows). In contrast, the sympathetic neuron marker tyrosine hydroxylase (TH) is undetectable in the dermis at E13.5 (Fig. 1D and G) and only minimally detected at E15.5 (Fig. 1E and H, open arrows). At E17.5, numerous TH^+^ sympathetic axons are observed along with sensory nerves (Fig. 1F and I, open arrows), and certain TH^+^ sympathetic axons intricately extend their branches towards blood vessels (Fig. 1F and I, yellow open arrows). These data clearly indicate that, after the establishment of co-branching between sensory nerves and blood vessels, sympathetic axons invade the dermis along with the pre-existing sensory nerves and extend their branches towards blood vessels. This sequential pattern of extension may signify the dependence of sympathetic axon trajectories on their association with pre-existing sensory projections (Wang et al., 2014).

### Sympathetic axons exhibit collateral branching, thereby extending their branches towards VSMC-covered blood vessels

**Fig. 2.**
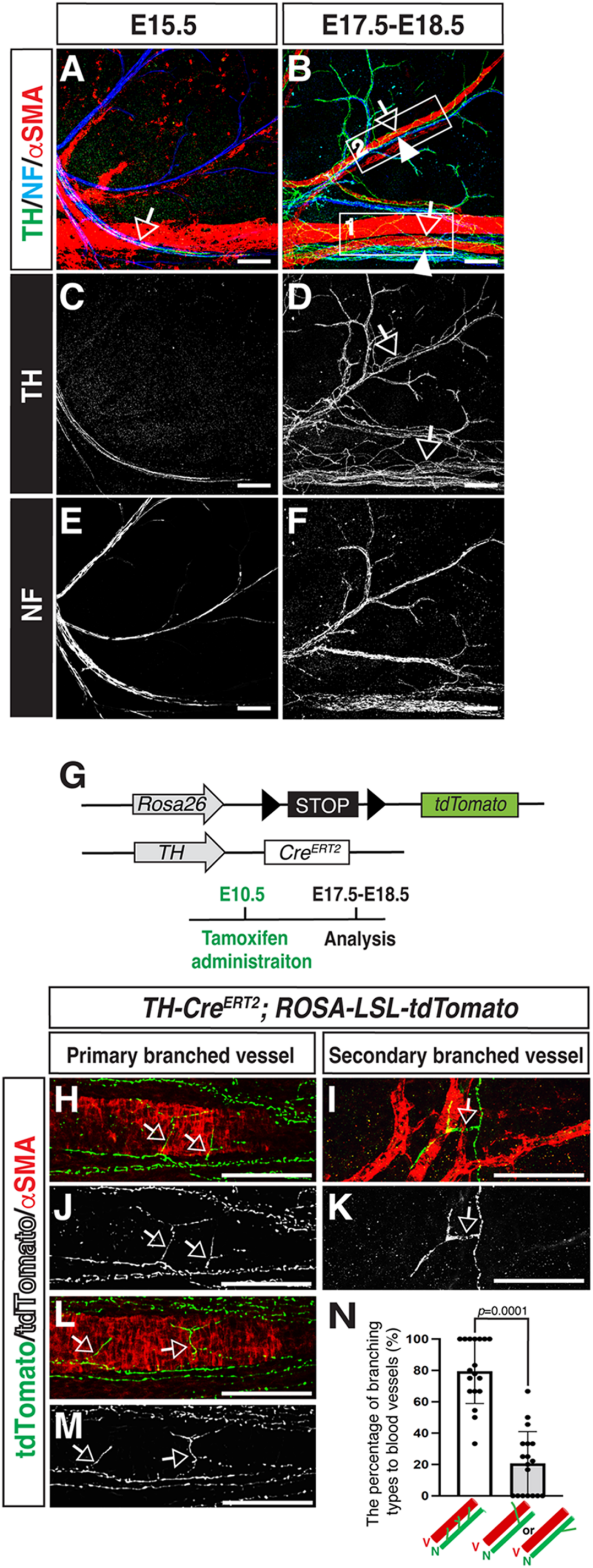
**Sympathetic axons innervate to smooth muscle cell covered blood vessels via collateral branching.** (A-F) Whole-mount triple immunofluorescence labeling of the dermis of the forelimb skin from E15.5 (A, C and E) and E17.5-E18.5 (B, D and F) wild-type embryos with antibodies to TH (green in A and B; white in C and D), NF (blue in A and B; white in E and F), and αSMA (red in A and B) is shown. The boxed region 1 and 2 in B indicates primary and secondary branched blood vessels, respectively. Open arrows indicate representative TH^+^/NF^+^ sympathetic axons and arrowheads indicate representative αSMA^+^ VSMC covered blood vessels. Scale bars: 100 µm. (G) Diagram illustrating the generation of *TH-Cre^ERT2^*; *ROSA-LSL-tdTomato* mice. The *Cre*-mediated excision activity was achieved by administering 1.5 mg tamoxifen by intraperitoneal injection (IP) at E10.5, and embryos were harvested at E17.5-E18.5 for whole-mount immunohistochemical analysis. (H-M) High resolution double immunofluorescence confocal microscopy of the dermis of the forelimb skin from *TH-Cre^ERT2^*; *ROSA-LSL-tdTomato* embryos with anti-dsRed antibody to detect tdTomato (green in H, I and L; white in J, K and M), together with anti-αSMA antibody (red in H, I and L). Open arrows indicate collateral branching of tdTomato^+^ individual sympathetic axons towards primary and secondary branched blood vessels. Scale bars: 100 µm. (N) Quantification of tdTomato^+^ individual sympathetic axons towards VSMC-covered blood vessels. Bars represent mean±s.e.m. Note that we quantified the number of TH^+^ sympathetic axon branches collaterally innervating blood vessels out of the total branches of sympathetic axons. There is a statistically significant difference, according to the nonparametric Mann–Whitney test. The data were collected from 18 limb skins (*n*=18). V: VSMC covered blood vessels. N: Sympathetic axons.

We next proceeded to meticulously examine the temporal patterns of sympathetic axon branching towards blood vessels in the dermis of the limb skin. In contrast to the E15.5 skin, the E17.5 skin exhibits TH^+^ sympathetic axon branching towards αSMA^+^ VSMCs of both primary and secondary branched blood vessels (Fig. 2A versus B, C versus D; open arrows; the box region 1 and 2 indicates primary and secondary branched blood vessels, respectively). A subset of TH^+^ sympathetic axons project their branches towards the hair follicles (Fig. S1A-C, outlined by dashed lines). To elucidate the branching patterns of individual sympathetic axons in the dermis of the limb skin, we employed a mosaic labeling technique on sympathetic axons. This method involved utilizing tamoxifen-inducible *TH-Cre^ERT2^* mice specific to sympathetic neurons (Rotolo et al., 2008), which were bred with *Cre*-dependent *ROSA-LSL-tdTomato* (Ai9) reporter mice (Madisen et al., 2010) (Fig. 2G). By administering low-dose tamoxifen injections at E10.5, individual sympathetic axons were discerned in the dermis of the limb skin from *TH-Cre^ERT2^*; *ROSA-LSL-tdTomato* embryos. Our observations revealed that the majority of sympathetic axons exhibit collateral branching, thereby extending their branches towards αSMA^+^ VSMC-covered primary and secondary blood vessels (Fig. 2 H-M, open arrows; N). Collectively, this data indicates that collateral branching mediates sympathetic axon branching towards VSMC-covered blood vessels.

### VSMCs express NGF during the stages when sympathetic axons extend their branches towards these VSMC-covered blood vessels

**Fig. 3.**
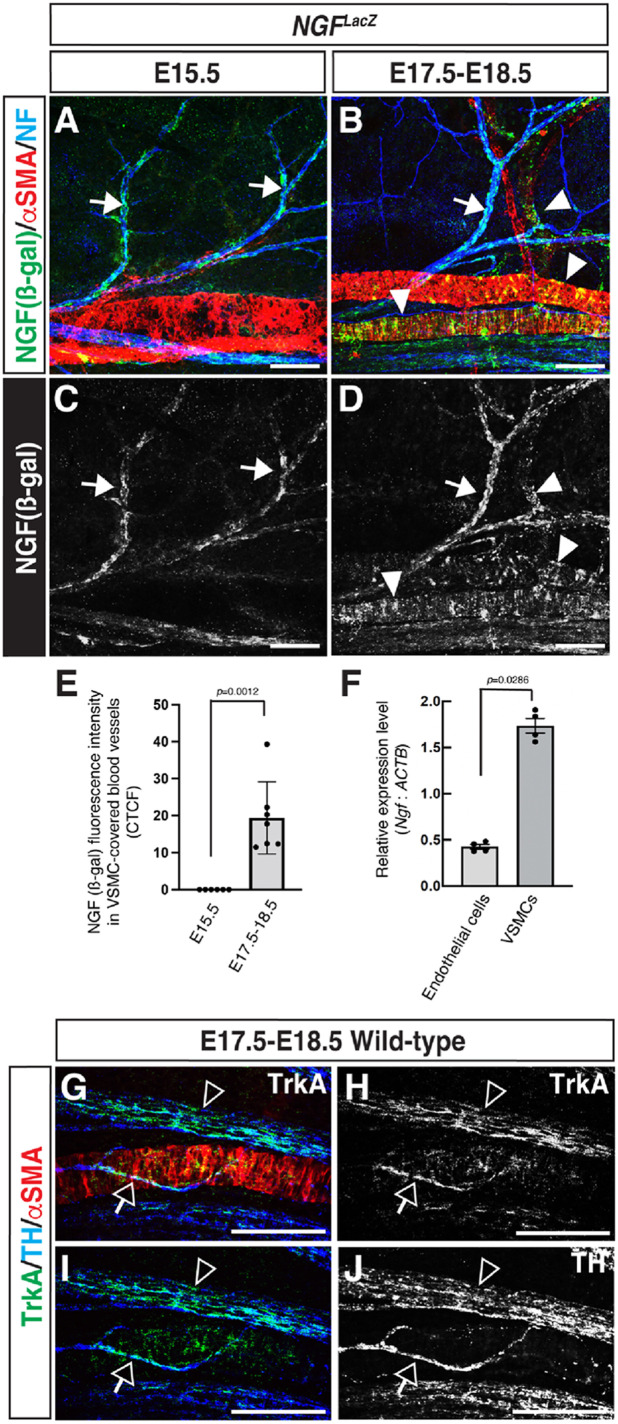
**VSMCs express NGF during the stages when sympathetic axons extend their branches towards these VSMC-covered blood vessels.** (A-D) Whole-mount triple immunofluorescence labeling of the dermis of the forelimb skin from E15.5 and E17.5-E18.5 *NGF^LacZ^* embryos with antibodies to ß-galactosidase (ß-gal) to detect NGF-LacZ (green in A and B; white in C and D), αSMA (red in A and B) and NF (blue in A and B). Arrows indicate NGF-expressing sensory nerves, while arrowheads indicate NGF-expressing VSMC-covered blood vessels. Scale bars: 100 µm. (E) Quantification of NGF expression in VSMC-covered blood vessels. The data were accomplished by measuring ß-gal fluorescence intensity using Fiji software and the unit is calculate with corrected total cell fluorescence (CTCF). All data were collected from three different littermates (*N*=3); the number of limb skins we analyzed is shown at each stage (*n*=6 in E15.5, *n*=7 in E17.5-E18.5). Bars represent mean±s.e.m. There is a statistically significant increase in NGF expression at E17-E18.5 compared to E15.5, according to the nonparametric Mann–Whitney test. (F) Relative *Ngf* mRNA expression levels in endothelial cells and VSMCs at E17.5-E18.5 limb skins were assessed by RT-qPCR (*n*=4). There is a statistically significant difference in *Ngf* expression between endothelial cells and VSMCs, according to the nonparametric Mann–Whitney test. (G-J) Whole-mount triple immunofluorescence labeling of the dermis of the forelimb skin from E17.5-E18.5 wild-type embryos with antibodies to the NGF receptor TrkA (green in G and I; white in H), TH (blue in G and I; white in J), and αSMA (red in G). Open arrows indicate a branched sympathetic axon innervating VSMC-covered blood vessel, while open arrowheads indicate sympathetic nerve bundle along with VSMC-covered blood vessel. Scale bars: 100 µm.

Nerve growth factor (NGF) is recognized for its role in promoting axon collateral branching, both during development and in the context of nervous system repair (Armijo-Weingart and Gallo, 2017; Diamond et al., 1987, 1992; Ketschek et al., 2015). NGF is also recognized for its role in regulating the extension of sympathetic axons towards peripheral targets, serving as a key factor that enhances nerve survival and elongation (Glebova and Ginty, 2004, 2005; Scott-Solomon et al., 2021). To address the spatial and temporal expression of NGF in the dermis of the limb skin, we analyzed *NGF^LacZ^* knock-in mice (Liu et al., 2012) and found that NGF is primarily expressed by sensory nerves at E15.5 (Fig. 3A and C, arrows), whereas both sensory nerves and VSMCs exhibit NGF expression at E17.5-E18.5, when sympathetic axons project their branches towards VSMC-covered blood vessels (Fig. 3B and D, arrows and arrowheads; E). This pattern of NGF expression in sensory nerves and VSMCs, along with sympathetic axon branching towards VSMC-covered blood vessels, was also observed in the dermis of the back skin at the same embryonic stages (Fig. S2A-E, arrows and arrowheads, NGF expression; S2F and G, open arrows, sympathetic axon branching). To define the NGF expression in endothelial cells (ECs) and VSMCs in the skin vasculature, we conducted fluorescence-activated cell sorting (FACS) to isolate ECs (CD45^−^/Ter119^−^/PECAM-1^+^/RFP^−^) and VSMCs (CD45^−^/Ter119^−^/PECAM-1^−^/RFP^+^) from the limb skin of E17.5-E18.5 *αSMA-Cre^ERT2^; R26R-RFP* embryos. Subsequently, we analyzed *Ngf* mRNA expression in these cells. Although *Ngf* expression is detectable in both FACS-isolated ECs and VSMCs, its expression is notably more abundant in VSMCs than in ECs (Fig. 3F). Considering that NGF and its receptor TrkA signaling promotes survival and innervation of sympathetic and sensory neurons (Belliveau et al., 1997), we observed the expression of TrkA in TH^+^ sympathetic axons, both those innervating VSMC-covered blood vessels and those innervating hair follicles in the skin (Fig. 3G-J, open arrows; Fig. S3A-D, open arrows). This data suggests that VSMC-derived NGF may potentially initiate the process of collateral branching in sympathetic axons directed towards these VSMC-covered blood vessels.

### *Ngf* deletion in VSMCs results in a reduction in collateral branching of sympathetic axons towards these VSMC-covered blood vessels

**Fig. 4.**
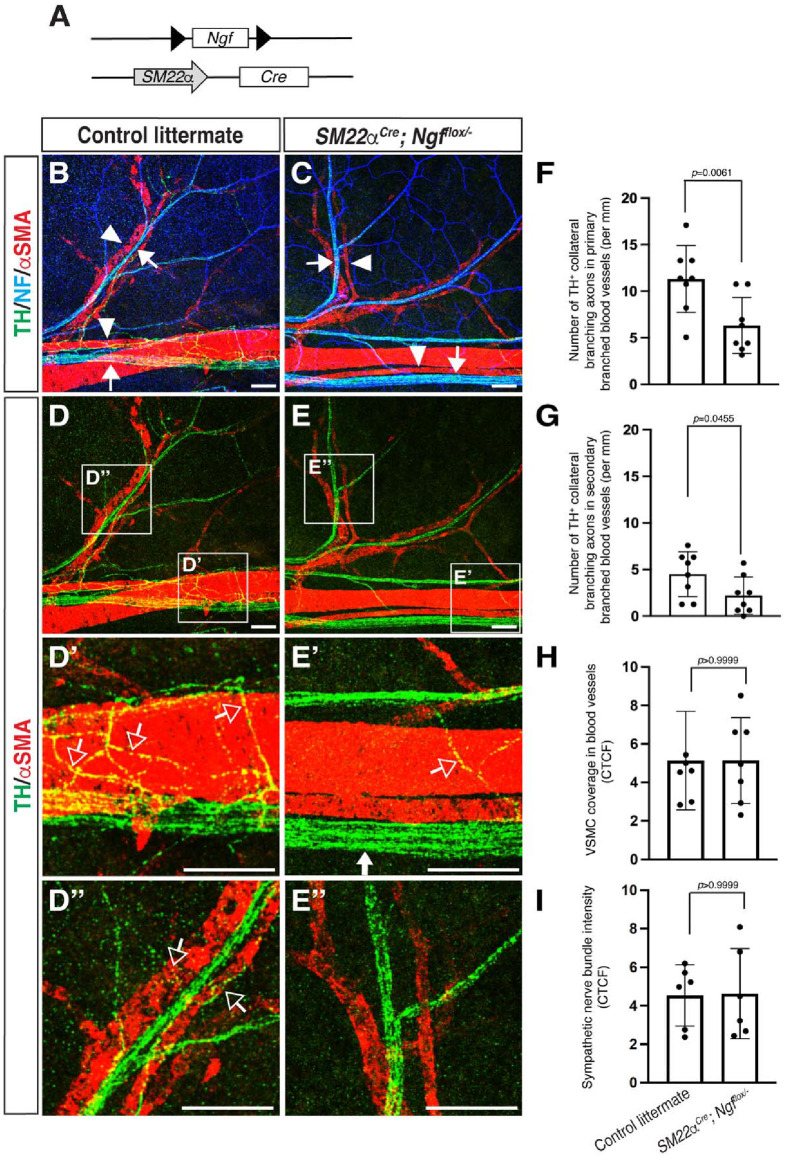
**VSMC-derived NGF is required for sympathetic collateral branching towards VSMC-covered blood vessels.** (A) Diagram illustrating the generation of *SM22α-Cre; Ngf^flox^*^/−^ mice. (B-E) Whole-mount triple immunofluorescence labeling of the dermis of the forelimb skin from E17.5-E18.5 *SM22αCre; Ngf^flox/−^* embryos and their control littermates with antibodies to TH (green), NF (blue), together with αSMA (red), is shown. The boxed regions in D and E are magnified in D′ and D″, and E′ and E″, respectively. Arrows indicate sensory nerve bundles and arrowheads indicate VSMC covered blood vessels. Open arrows indicate sympathetic axon branching towards VSMC-covered blood vessels. Scale bars: 100 µm. (F-G) Quantification in the total number of TH^+^ sympathetic collateral branching to VSMC-covered primary and secondary branched blood vessels (*N*=4 per genotype, eight limb skins were analyzed for each genotype; *n*=8). Bars represent mean±s.e.m. There is a statistically significant difference, according to the nonparametric Mann–Whitney test. (H-I) Quantification of VSMC coverage in blood vessels (H) and the intensity of sympathetic nerve bundles (I) were achieved by measuring fluorescence intensity using Fiji software and the unit is calculate with corrected total cell fluorescence (CTCF). The data were collected from four different littermates per genotype (*N*=4) and the number of limb skins we analyzed for each genotype is shown (*n*=6). Bars represent mean±s.e.m. There is no significant difference, according to the nonparametric Mann–Whitney test.

To address whether VSMC-derived NGF is necessary for establishing sympathetic axon branching towards blood vessels in the dermis of the limb skin, we analyzed the patterns of sympathetic axon branching in VSMC-specific *Ngf* conditional knockout mice (Tampakakis et al., 2021), using VSMC-specific *SM22α-Cre* mice (Holtwick et al., 2002) and floxed *Ngf* (*Ngf^floxl/−^*) mice (Muller et al., 2012) (Fig. 4A). VSMC-specific *Ngf* conditional knockout embryos exhibit very few sympathetic axons associated with VSMC-covered primary and secondary blood vessels in the dermis of the limb skin at E17.5-E18.5 compared to control littermates (Fig. 4D versus E, D′ versus E′, D″ versus E″, open arrows; F and G). In contrast, the co-branching of sensory nerve bundles and VSMC-covered blood vessels was not affected (Fig. 4B versus C, arrows and arrowheads). This compromised collateral branching of sympathetic axons is unlikely to result from abnormal VSMC coverage in blood vessels (Fig. 4H) or a reduction in sympathetic nerve bundles (Fig. 4I). Thus, VSMC-derived NGF is required for the initiation of collateral branching in sympathetic axons directed towards these VSMC-covered blood vessels.

## DISCUSSION

Our findings are fully consistent with the previous *in vitro* studies that have shown the significance of NGF's local action in promoting axon collateral branching (Armijo-Weingart and Gallo, 2017; Ketschek et al., 2015). Our *in vivo* studies demonstrate that VSMC-derived NGF in blood vessels is important to induce the formation of sympathetic collateral branching, specifically for their innervation of blood vessels. Given that after birth in mice, the axon guidance molecule netrin-1 serves as a VSMC-derived secreted factor for facilitating sympathetic innervation of VSMC-covered blood vessels in the skin vasculature (Brunet et al., 2014), NGF and netrin-1 may mediate sequential steps in sympathetic innervation of blood vessels.

The observation of VSMCs expressing NGF during the stages when sympathetic axons extend their branches towards these VSMC-covered blood vessels poses an intriguing question about mechanisms governing the spatial and temporal expression of NGF. It is generally analogous to our previous studies demonstrating the spatiotemporal changes in NGF expression by venous and arterial VSMCs within coronary vessels, crucial for the sympathetic innervation of the heart (Nam et al., 2013). Given the precise regulation of NGF expression in a cell-type-specific manner during development, there is a need for further investigation into molecular mechanisms that govern the transcriptional regulation of the *Ngf* gene in VSMCs.

**Fig. 5.**
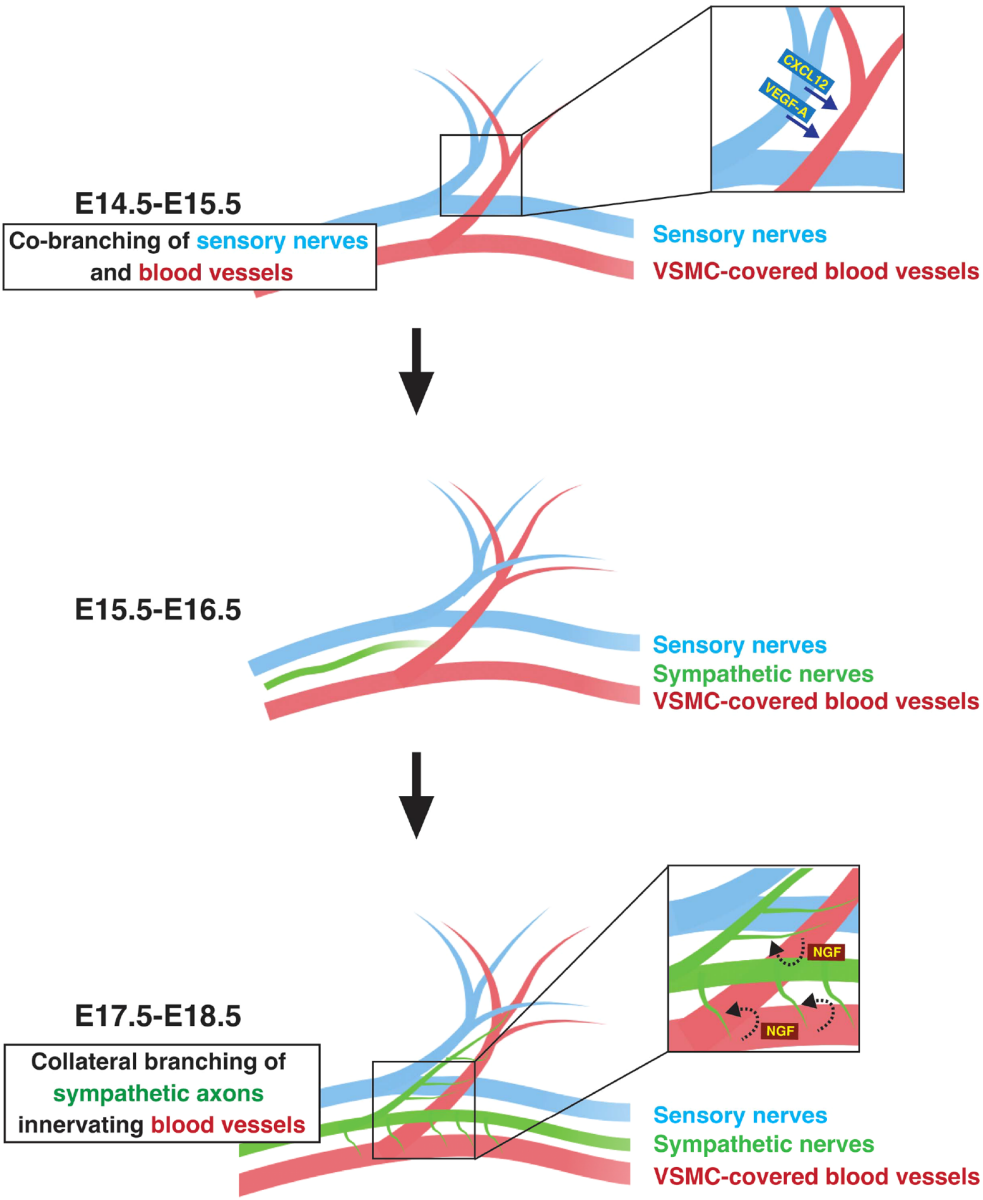
**Schematic model illustrating the co-branching of sensory nerves and blood vessels, followed by vascular innervation by sympathetic axons in the embryonic skin.** Our previous studies have revealed that CXCL12 and VEGF-A serve as sensory nerve-derived signals for the development of co-branching between sensory nerves and VSMC-covered blood vessels (Li et al., 2013; Mukouyama et al., 2005, 2002). In this study, we demonstrate that VSMC-derived NGF induces collateral branching of sympathetic axons, facilitating their innervate into VSMC-covered blood vessels. These illustrations were created with BioRender.com.

Lastly, our *in vivo* studies presented here focus on the embryonic skin vasculature model, where we illustrate (1) the collateral branching of sympathetic axons towards VSMC-covered blood vessels, and (2) the critical role played by VSMC-derived NGF in inducing sympathetic axon branching to innervate these blood vessels (Fig. 5). The question of whether NGF derived from VSMCs plays an essential role in inducing sympathetic axon branching to innervate VSMC-covered blood vessels in other organ vasculature remains to be addressed. Indeed, previous studies demonstrated the development of sympathetic innervation in the postnatal mesenteric vasculature model. These investigations revealed that netrin-1 functions as a VSMC-derived secreted factor, facilitating sympathetic innervation of VSMC-covered blood vessels in postnatal mesenteric vessels (Brunet et al., 2014). To explore the potential involvement of NGF derived from VSMCs in sympathetic innervation of VSMC-covered blood vessels, including mesenteric vasculature, it is essential to examine the expression of NGF in VSMC-covered vessels during developmental and postnatal stages. Moreover, it is crucial to assess the impact on sympathetic axon branching towards VSMC-covered blood vessels in various organ vasculature using VSMC-specific *Ngf* conditional knockouts.

## MATERIALS AND METHODS

### Mice

All animal procedures were approved by the National Heart, Lung, and Blood Institute (NHLBI) Animal Care and Use Committee in accordance with NIH research guidelines for the care and use of laboratory animals. The following mice were used in this study: C57BL/6J mice (The Jackson Laboratory), *TH-Cre^ERT2^* mice (The Jackson Laboratory, stock number 008532) (Rotolo et al., 2008), *ROSA-LSL-tdTomato* (Ai9) mice (The Jackson Laboratory, stock number 007905) (Madisen et al., 2010), *αSMA-Cre^ERT2^* mice (Wendling et al., 2009) and *NGF^LacZ^* knock-in mice (Liu et al., 2012). E17.5-E18.5 *SM22α-Cre; Ngf^flox^*^/−^ and littermate control embryos (Tampakakis et al., 2021) were kindly provided by Dr Emmanouil Tampakakis (Johns Hopkins University). *αSMA-Cre^ERT2^; R26R-RFP* mice were kindly provided by Dr Thomas Arnold (UCSF). The *Cre*-mediated excision was achieved by administering tamoxifen (Sigma-Aldrich, T-5648) by intraperitoneal injection (I.P.).

### Whole-mount immunohistochemistry of embryonic skin

Staining was performed essentially as described previously (Li and Mukouyama, 2011). In brief, embryonic forelimb skin were collected and fixed by 4% paraformaldehyde at 4°C overnight. After being washed in PBS the next day, the samples were incubated with primary antibodies in PBS-Triton X-100 solution with 10% heat inactivated goat or donkey serum buffer at 4°C for overnight. The primary antibodies used are: rabbit anti-TH antibody (Millipore/Sigma, AB152; 1:250) to detect sympathetic nerves; rabbit anti-CGRP antibody (Millipore/Sigma, PC205L; 1:500) to detect sensory nerves; chicken anti-neurofilament (NF, Abcam, AB4680; 1:1000) or mouse anti-β-tubulin (βIII) (clone Tuj1, Biolegend, 801202; 1:500) antibodies to detect peripheral nerves; rabbit anti-ß-galactosidase antibody (ß-gal, Cappel/MP Biomaterials, SKU0856032; 1:500) to detect LacZ indicating nerve growth factor (NGF); goat anti-TrkA antibody (R&D, AF1056; 1:200) to detect TrkA receptor; rabbit anti-dsRed antibody to detect tdTomato (Clontech/Takara, 632496; 1:1000); Armenian hamster anti-PECAM-1 antibody (Miliipore, MAB1398Z; 1:250) to detect endothelial cells and Cy3 or FITC-conjugated anti-αSMA antibody (clone1A4, Millipore/Sigma, C6198; 1: 1:500) to detect smooth muscle cells; Chick anti-Keratin 14 antibody (K14, Millipore/Sigma, SIG-3476-100; 1:200) to detect hair follicles. For immunofluorescence detection, either Cy3-, Alexa-488-, Alexa-568-, Alexa-647-, Alexa 633-conjugated secondary antibodies (Jackson ImmunoResearch 127-605-160 or 115-605-206; or ThermoFisher Scientific, A11041, A11039 or A21103; 1:250, 1 h at room temperature) were used. All stained samples were mounted with ProLong Gold Antifade Mounting solution (ThermoFisher Scientific). All confocal microscopy was carried out on a Leica TCS SP5 confocal microscope (Leica). Quantification of NGF expression, VSMC coverage, and the intensity of sympathetic nerve bundles was achieved by measuring fluorescence intensity using Fiji software. The unit was calculated with corrected total cell fluorescence (CTCF) as the mean fluorescence value after subtracting the background signals. Number of embryos is indicated as ‘n’ in figure legends. Statistical significance of samples was assessed using the nonparametric Mann–Whitney test and graphs were created with Prism 9 software. Note that a novel imaging transformer model (A.R., H.X., C.C., manuscript in preparation) was developed to enhance the image quality of the confocal microscope scans for the single axon tracing images shown in Fig. 2. This model was first pre-trained on a large-curated database and further fine-tuned on the data of this study. The structured similarity index was optimized to restore the image quality over the paired low- and high-quality data. The trained model was applied to acquired images on a NVIDIA RTX A6000 GPU, with the inference time being about 5 min*.* To perform quantification measurements of branched TH^+^ single axons, we initially defined an image area in 10× confocal z-stack images of whole-mount limb skin from E17.5 embryos. This was achieved by using the positions of large-diameter blood vessels covered by *α*SMA^+^ vascular smooth muscle cells (VSMC) as a frame of reference. The images presented in Figs 2B and 4B-E are representative examples of the defined images. We then quantified the total number of branched TH^+^ single axons directed towards VSMC-covered blood vessels in the defined image area of each limb skin, normalizing them by the total lengths of the measured blood vessels within the defined image area.

### Flow cytometry

Forelimb skins were peeled off from E17.5-E18.5 embryos of *αSMA-Cre^ERT2^; R26R-RFP* mice in HBSS (Gibco, 14025) and dissociated by digestion with 0.1% type I collagenase (Worthington, LS004194), 0.3% Dispase (Gibco, 17105-041), 0.05% deoxyribonuclease type 1 (DNase 1; Millipore/Sigma, D4527) and 5% fetal bovine serum (FBS, Hyclone, SH3007) in L15 medium (ThermoFisher Scientific, 21083) at 37°C for 1 h. The dissociated cells were filtered through 100-μm filters and washed with FACS buffer (1% BSA; Millipore/Sigma, A-9418), 0.1 M HEPES (ThermoFisher Scientific, 21083-027), 1x Pen-Strep (Gibco, 15140), 0.025% DNase I, in L15 medium). Cells were then incubated with FITC-conjugated anti-PECAM-1 antibody (1:50, BD Pharmingen, 553372) to detect endothelial cells. To eliminate erythrocytes and myeloid cells, the dissociated skin cells were incubated with APC-conjugated anti-Ter119 (eBioscience, 17-5921-83, 1:100) and anti-CD45 (eBioscience, 17-0451-83, 1:100) together with FITC-conjugated anti-PECAM-1 antibody for 30 min on ice. Cell viability was assessed using 7-aminoactinomycin D (ThermoFisher Scientific, A1310). The CD45^−^Ter119^−^/PECAM-1^+^/RFP^−^ endothelial cells and CD45^−^Ter119^−^/PECAM-1^−^/RFP^+^ VSMCs populations were sorted using BD FACSAria-IIu cell sorter with BDFACSDiva software (BD Science).

### RNA isolation, reverse transcription (RT)-PCR and RT-quantitative PCR (RT-qPCR)

mRNA was purified from sorted cells by RNeasy mini kit (Qiagen, 74104) according to the manufacturer’s instructions. Genomic DNA was eliminated with DNase I and RNase inhibitor treatment at 37°C for 20 min. cDNA was reverse transcribed from mRNA using ReverTra Ace qPCR RT Mater Mix (Toyobo, FSQ-301). RT-qPCR was performed with Thunderbird Next SYBR qPCR Mix (Toyobo, QPX-201) on LightCycler 96 (Roche). Relative quantification of *Ngf* expression level was obtained by normalizing against *ACTB* transcript abundance. The sequence of the RT-qPCR primers are as follows: *Ngf* forward: 5′-GTT TTG CCA AGG ACG CAG CTT TC-3′ and *Ngf* reverse: 5′-GTT CTG CCT GTA CGC CGA TCA A-3′. *ACTB* forward: GGA CAT CCG CAA AGA CCT GTA-3′ and *ACTB* reverse: 5′-GCT CAG GAG GAG CAA TGA TCT-3′.

## Supplementary Material

10.1242/biolopen.060147_sup1 Supplementary information
