# Supplementary material for: Vascular smooth muscle cell-derived nerve growth factor regulates sympathetic collateral branching to innervate blood vessels in embryonic skin
Source: Biol Open. 2024 May 21;13(5):bio060147. doi: 10.1242/bio.060147 (PMC11139032; doi:10.1242/bio.060147)
Supplement: Supplementary information [file biolopen-13-060147-s1.pdf]

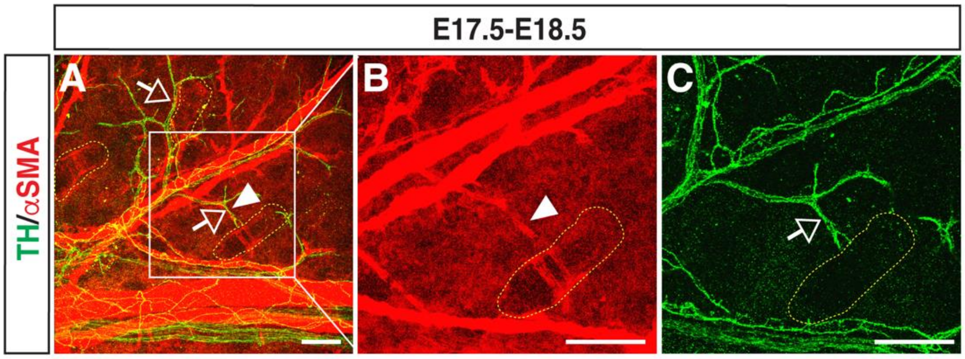

Fig. S1. Certain TH$^+$ sympathetic axons project their branches towards the hair follicles.

Whole-mount double immunofluorescence labeling of the dermis of the forelimb skin with antibodies to TH (green in A and C) and αSMA (red in A and B). Note that αSMA staining detects both VSMC-covered blood vessels (indicated by arrowheads) and a segment of hair follicles (outlined by dashed lines). Open arrows indicate TH$^+$ sympathetic axons branching towards hair follicles. Scale bars represent 100 μm.

Biology Open • Supplementary information

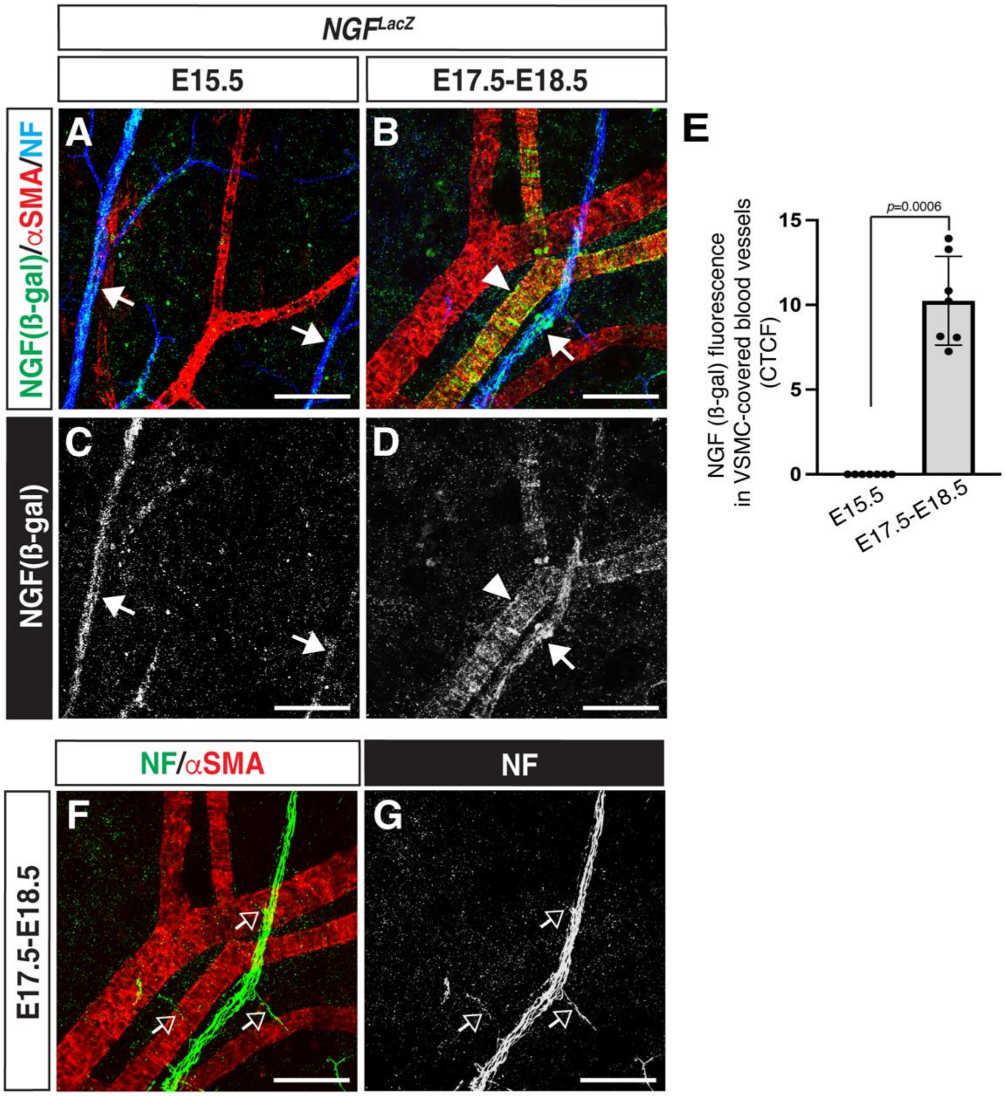

Fig. S2. VSMC-covered blood vessels express NGF in the dermis of the back skin. (A-D) Whole-mount triple immunofluorescence labeling of the dermis of the back skin from E15.5 and E17.5-E18.5 *NGF*^{LacZ} embryos with antibodies to ß-gal to detect NGF-LacZ (green in A and B; white in C and D), αSMA (red in A and B), and NF (blue in A and B). Arrows indicate NGF-expressing sensory nerves, while arrowheads indicate NGF-expressing VSMC-covered blood vessels. Scale bars represent 100 µm.

Biology Open • Supplementary information

(E) Quantification of NGF expression in VSMC-covered blood vessels. The data was collected from 3 different littermates (N=3); the number of back skins we analyzed is shown at each stage (n=7). Bars represent mean ± SEM. There is a statistically significant increase in NGF expression at E17.5-E18.5 compared to E15.5 ($p<0.001$), according to the nonparametric Mann-Whitney test.

(F-G) Whole-mount triple immunofluorescence labeling of the dermis of the back skin from E17.5-E18.5 embryos with antibodies to αSMA (red in F) and NF (green in F; white in G). Open arrows indicate sympathetic axon branching towards VSMC-covered blood vessels. Scale bars represent 100 μm.

Biology Open • Supplementary information

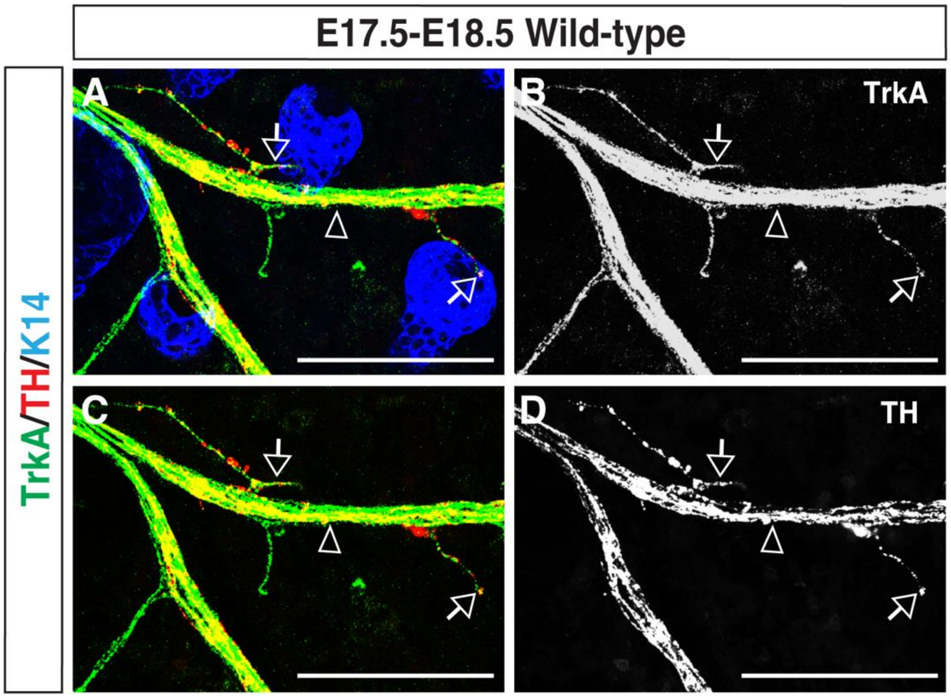

Fig. S3. Both TH^{+} sympathetic nerve bundles and axons innervating the hair follicles express TrkA.

Whole-mount triple immunofluorescence labeling of the dermis of the forelimb skin from E17.5-E18.5 wild-type embryos with antibodies to TrkA (green in A and C; white in A), TH (red in A and C; white in D), and Keratin 14 (K14, blue in A). Open arrows indicate TH^{+}/TrkA^{+} sympathetic axon branching towards K14^{+} hair follicles, while open arrowheads indicate TH^{+}/TrkA^{+} sympathetic nerve bundles. Scale bars represent 100 μm.

Biology Open • Supplementary information
